# Mediating role of brain aging in the effect of white matter hyperintensities on post-stroke aphasia severity

**DOI:** 10.3389/fnagi.2025.1629870

**Published:** 2025-10-16

**Authors:** Guihua Xu, Yongsheng Wu, Rui Zhu, Junyu Qu, Wenwen Xu, Jiaxiang Xin, Dawei Wang

**Affiliations:** ^1^Department of Radiology, Qilu Hospital of Shandong University and Qilu Medical Imaging Institute of Shandong University, Jinan, China; ^2^MR Research Collaboration, Siemens Healthineers Ltd., Shanghai, China; ^3^Shandong Key Laboratory for Magnetic Field-free Medicine and Functional Imaging, Institute of Magnetic Field-free Medicine and Functional Imaging, Shandong University, Jinan, China; ^4^National Innovation Platform for Industry-Education Integration in Medicine-Engineering Interdisciplinary, Shandong University, Jinan, China

**Keywords:** post-stroke aphasia, white matter hyperintensities, brain age, interaction analysis, mediation analysis

## Abstract

**Objectives:**

White matter hyperintensities (WMH) have been associated with the severity of post-stroke aphasia (PSA), but the contribution of overall brain health remains unclear. Brain age is a neurobiological indicator of aging that is based on whole-brain structural neuroimaging. This study investigated the impact of brain age on language function after stroke.

**Methods:**

Fifty-seven patients with PSA and left-hemisphere lesions were included. The Fazekas scale was used to evaluate WMH burden, including periventricular WMH (PWMH) and deep WMH (DWMH). Brain age was estimated using structural 3D T1-weighted imaging, and the Brain-Predicted Age Difference (brain-PAD) was calculated. Multivariate linear regression and mediation analysis were conducted to examine associations among WMH burden, brain-PAD, and aphasia severity. The interaction between WMH burden and brain-PAD was also assessed.

**Results:**

Higher levels of PWMH and DWMH were associated with increased brain-PAD in PSA patients (PWMH: *p* = 0.024; DWMH: *p* < 0.001). Mediation analysis indicated that WMH had an indirect effect on auditory comprehension via brain-PAD (PWMH: *β* = −9.360, *p* = 0.028, *q* = 0.042) and a direct effect on naming impairment (PWMH: *β* = −15.812, *p* = 0.030, *q* = 0.042; DWMH: *β* = −19.217, *p* = 0.030, *q* = 0.042). A significant interactive effect of PWMH burden and brain-PAD on auditory comprehension was also observed (*β* = −4.040, *p* = 0.004, *q* = 0.033).

**Conclusion:**

Our findings highlight the influence of neuroanatomical aging and WMH burden on post-stroke language deficits, supporting the consideration of both brain-PAD and WMH severity when assessing aphasia severity to inform clinical assessment and treatment planning.

## 1 Introduction

White matter hyperintensities (WMH) are typical imaging markers of cerebral small vessel disease, characterized by hyperintensity on T2-weighted or fluid-attenuated inversion recovery (FLAIR) images in the periventricular and/or deep white matter regions (Fazekas et al., 1987; Kim et al., 2008). The prevalence of WMH increases with age, particularly among individuals aged > 60 years (de Leeuw et al., 2001; Van Leijsen et al., 2017). Although WMH has historically been considered a benign feature of normal aging, there is increasing evidence that it serves as an important indicator of compromised brain health (Lau et al., 2017; Lin et al., 2017). It is closely associated with conditions such as hypertension, diabetes, dyslipidemia, and atherosclerosis (Longstreth et al., 1996). Additionally, WMH has been linked to various neurological deficits, including cognitive decline and depression (Garde et al., 2000; Dalby et al., 2010).

Several investigations have shown that a greater WMH burden is associated with worse prognosis in post-stroke aphasia (PSA); affected patients exhibit more severe language deficits and diminished rehabilitation outcomes (Wright et al., 2018; Vadinova et al., 2023). However, recent findings have revealed substantial heterogeneity in the impact of WMH on aphasia recovery, such that some patients maintain relatively preserved language function despite severe WMH (Brickman et al., 2011; Basilakos et al., 2019). This heterogeneity suggests that WMH does not influence language function solely through direct pathological effects but may be modulated by additional factors.

Brain age, a neuroimaging-based biomarker, offers a novel approach to evaluating brain health (Cole and Franke, 2017). By comparing an individual’s neuroimaging data with population-based reference datasets, brain age estimates the biological age of the brain, thus reflecting the extent of brain aging (Baecker et al., 2021). The difference between brain age and chronological age, termed Brain-Predicted Age Difference (brain-PAD), indicates whether biological aging is occurring more rapidly or slowly than expected (Chakrabarty et al., 2023). This metric has been widely utilized in studies of neurological and psychiatric disorders, including Alzheimer’s disease (Lee et al., 2022), Parkinson’s disease (Chen et al., 2024), neuronal intranuclear inclusion disease (Zhu et al., 2024), and schizophrenia (Abram et al., 2023).

Previous studies have independently examined associations between WMH burden and brain-PAD, as well as between brain-PAD and PSA severity. For example, the correlation between increased brain age and performance in semantic tasks (Busby et al., 2022; Wrigglesworth et al., 2022). However, mechanisms underlying the interactions among these three factors require clarification. Specifically, the extent to which brain-PAD mediates or moderates the relationship between WMH burden and PSA severity, and whether synergistic effects of WMH and brain-PAD on language impairment exist, have yet to be systematically investigated.

This study sought to systematically elucidate the relationship among WMH burden, brain-PAD, and aphasia severity in patients with PSA. The analysis addressed three principal questions: (1) the association between WMH burden and brain-PAD in patients with PSA; (2) whether brain-PAD functions as a mediating factor in the relationship between WMH burden and language impairment; and (3) the interactive effects of periventricular (PWMH) and deep WMH (DWMH) with brain-PAD on specific aphasia subdomains. Through these analyses, the study aimed to establish a multidimensional analytical framework to clarify the complex etiology of PSA and to identify novel therapeutic targets for personalized neurorehabilitation strategies.

## 2 Materials and methods

### 2.1 Participants

**Figure 1:**
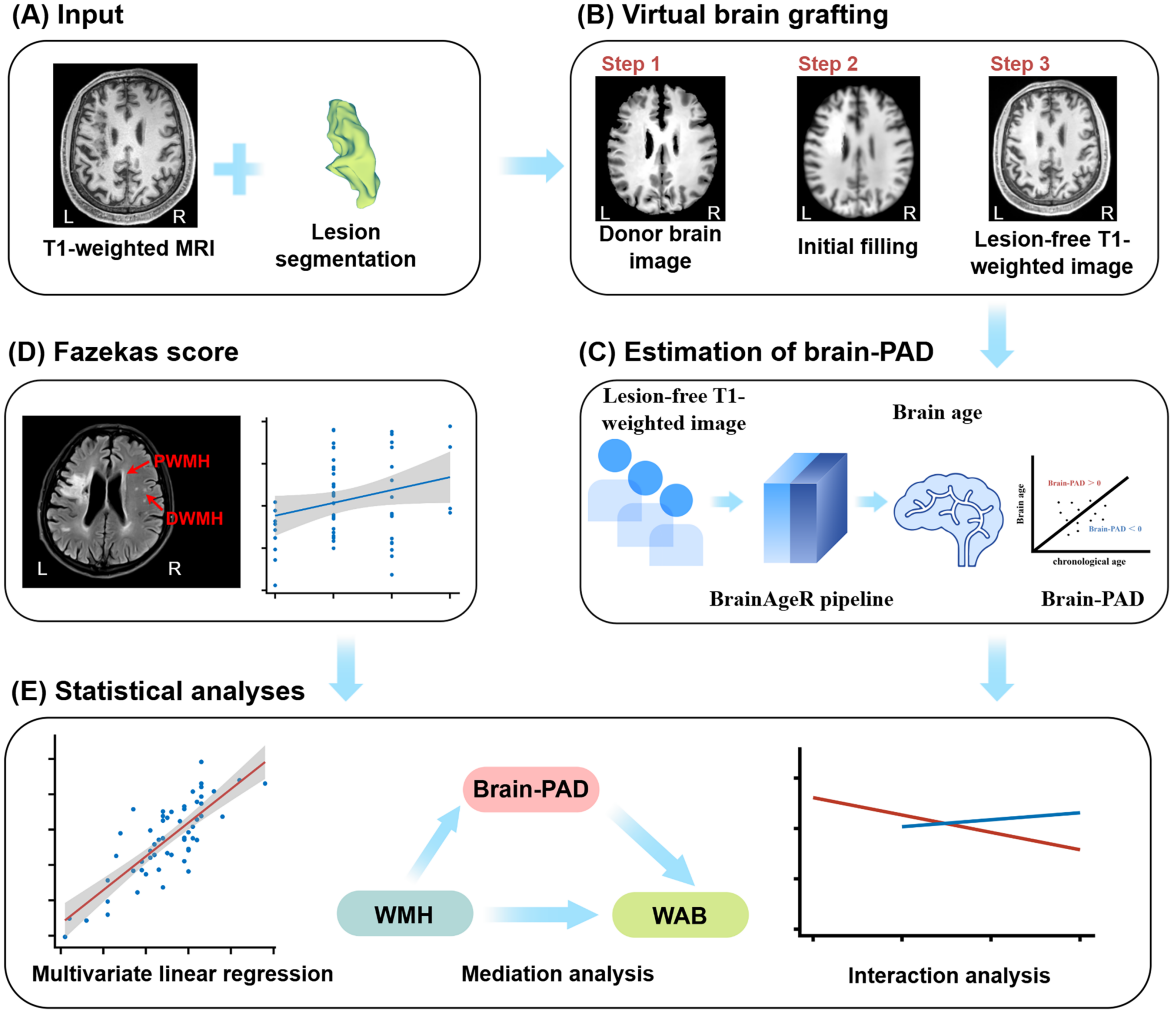
Overview of the analytical approach. **(A)** T1WI image and lesion mask of a patient with PSA. **(B)** Workflow of VBG. **(C)** Estimation of brain-PAD. **(D)** Classification of WMH using the Fazekas score, distinguishing PWMH and DWMH. **(E)** Summary of statistical analyses performed, including multiple linear regression, mediation analysis, and interaction analysis. Brain-PAD, Brain-Predicted Age Difference; DWMH, Deep White Matter Hyperintensities; PSA, Post-Stroke Aphasia; PWMH, Periventricular White Matter Hyperintensities; VBG, Virtual Brain Grafting; WMH, White Matter Hyperintensities.

This study was approved by the Ethics Committee of Qilu Hospital of Shandong University (Approval No.: KYLL-202404-036). Written informed consent was obtained from all participants or their legal guardians prior to enrollment. In total, 57 patients with PSA were recruited based on the following inclusion criteria: (i) first-ever stroke involving the left hemisphere, confirmed by cranial computed tomography or magnetic resonance imaging (MRI); (ii) persistent aphasia from the first day after stroke onset; (iii) entry into the chronic recovery phase (more than 6 months after the stroke); (iv) native Mandarin speaker; and (v) right-handedness. Exclusion criteria were: (i) history of other neurological disorders; (ii) history of severe head trauma; (iii) poor-quality MRI images unsuitable for analysis; and (iv) contraindications to MRI examination. Figure 1 summarizes an overview of the analytical approach.

### 2.2 Neuroimaging acquisition

All MRI data were acquired using a 3 T scanner (MAGNETOM Prisma; Siemens Healthcare, Erlangen, Germany) equipped with a 64-channel head coil. All patients underwent 3D T1-Weighted Imaging (T1WI) sagittal high-resolution and T2-weighted Fluid-attenuated Inversion Recovery (T2-FLAIR) sequences. 3D T1WI images were collected with an MPRAGE sequence and the following parameters: repetition time (TR) = 1,610 ms, echo time (TE) = 2.23 ms, flip angle = 8°, field of view (FOV) = 224 × 224 mm^2^, GRAPPA acceleration factor = 2 (Griswold et al., 2002), voxel size = 1.0 × 1.0 × 1.0 mm^3^, inversion time (TI) = 900 ms, bandwidth = 200 Hz/Px, and total acquisition time = 3 min 20 s. T2-FLAIR images were acquired in the axial plane using fast spin-echo sequence with the following parameters: TR/TE = 7500/95 ms, FOV = 230 × 223 mm^2^, slice thickness = 5 mm, TI = 2,298 ms, number of slices = 24, bandwidth = 287 Hz/Px, GRAPPA acceleration factor = 2, and total acquisition time = 1 min 47 s.

### 2.3 Lesion delineation

The open-source software 3D Slicer^1^ was used to manually delineate stroke lesion masks on 3D-T1WI. Stroke lesion volumes were calculated in native space for subsequent analyses. T2-FLAIR images were used to verify lesion location.

### 2.4 Estimation of brain-PAD

T1WI images were processed using Virtual Brain Grafting (VBG), a fully automated open-source workflow (Radwan et al., 2021). The VBG method segments brain tissue by combining the lesion mask with the patient’s intact hemisphere. Specifically, the lesioned area was reconstructed through mirroring the intact hemisphere, creating a lesion-free T1WI image for subsequent processing (Yourganov et al., 2016; Salvalaggio et al., 2020; Kristinsson et al., 2021).

To predict brain age, we processed lesion-free T1WI images using the BrainAgeR pipeline (Cole and Franke, 2017; Cole et al., 2017, 2018). This pipeline employs a cohort-based machine learning model that was trained on data from 3,377 healthy individuals aged 18 to 92 and validated on 857 individuals. The model predicted chronological age with a mean absolute error < 5 years, accounting for 94.6% of the variance in chronological age. The procedure included the following steps: (1) segmentation of T1WI images into gray matter, white matter, and cerebrospinal fluid; (2) nonlinear spatial alignment and normalization using the DARTEL toolbox; (3) quality control using the modified FSL slicesdir tool; (4) cerebrospinal fluid was removed, and probabilistic tissue in gray matter and white matter was vectorized, combined, and subjected to principal component analysis (PCA) to reduce data complexity, retaining those that explained 80% of variance; and (5) prediction of brain age using a Gaussian regression model implemented in the Kernlab R package.

The degree of brain aging was quantified by calculating the difference between predicted brain age and chronological age (i.e., brain-PAD). A positive brain-PAD value indicated that the predicted brain age surpasses the individual’s chronological age, signifying advanced brain aging, whereas a negative value reflected that the individual’s chronological age exceeds their predicted brain age, indicating delayed brain aging.

### 2.5 WMH assessment

WMH burden was evaluated using the Fazekas scale by two radiologists based on T2-FLAIR images (Fazekas et al., 1987). Intraclass correlation coefficients (ICC) were calculated to assess inter-rater reliability (Supplementary Table 1) (Koo and Li, 2016). In cases of disagreement, a consensus was reached to determine the final score. WMH was graded on the right hemisphere in accordance with the principle of hemispheric symmetry (Wright et al., 2018; Basilakos et al., 2019). For PWMH, a score of 0 indicated no lesion; 1, a thin lesion; 2, a smooth halo pattern; and 3, involvement of deep white matter. For DWMH, a score of 0 denoted no lesion; 1, punctate foci; 2, small confluent areas; and 3, large confluent areas.

### 2.6 Language assessment

Language function was assessed using the Western Aphasia Battery (WAB) (Shewan and Kertesz, 1980; Li et al., 2023), which includes subtests for spontaneous speech, repetition, naming, and auditory comprehension. Each subtest contributes to the total score, which is aggregated to yield the Aphasia Quotient (AQ). The AQ serves as an index of aphasia severity, ranging from 0 to 100; lower scores indicate more severe language impairment.

### 2.7 Statistical analyses

All statistical analyses were conducted in the R software environment (v4.0.3). The threshold for statistical significance was set at *p* value < 0.05. Given the potential type I errors from multiple comparisons, we additionally reported *q* values (*p* values adjusted for FDR using the Benjamini-Hochberg procedure) to ensure the reliability of statistical inferences. To comprehensively examine the relationship between WMH burden and brain-PAD, and to assess whether WMH influences aphasia severity through brain-PAD, the following steps were undertaken:

#### 2.7.1 Multivariate linear regression analysis

We assess the association between brain-PAD and WMH. Chronological age, sex, education level, hypertension, diabetes mellitus, lesion volume, and time since stroke were included as covariates.

#### 2.7.2 Mediation analysis

To assess whether brain-PAD mediates the effect of WMH on aphasia severity, we adopted the three-step mediation analysis framework proposed by Baron and Kenny (1986). Step 1: The direct effect of WMH on aphasia severity was tested. Step 2: The effect of WMH on brain-PAD was examined, as established in the preceding regression analysis. Step 3: The effect of WMH on aphasia severity was re-evaluated while controlling for brain-PAD. The “lavaan” package in R was used to estimate 95% confidence intervals and standard errors via bootstrapping with 1,000 resamples. To quantify the magnitude of the mediating effect, the effect size was calculated as the ratio of the indirect effect to the total effect of WMH on aphasia severity.

#### 2.7.3 Interaction analysis

To test the hypothesis that PWMH and DWMH affect distinct dimensions of language performance, we constructed a multiple regression model that includes an interaction analysis. Each WAB subscale was used as the dependent variable; PWMH/DWMH and brain-PAD were entered as independent variables. Interaction terms were included, and their statistical significance was tested to evaluate the moderating effects of brain-PAD on the relationship between WMH burden and language outcomes. The effect size was calculated using Cohen *f* ^2^ (Cohen, 2009). To intuitively illustrate the interaction effect, the total sample (*n* = 57) was evenly divided into three tertile groups based on brain-PAD values, with 19 participants in each group, resulting in 3 groups: (1) A “delayed” group, which included individuals whose brain-PAD values fell within the lowest tertile. (2) An “age-congruent” group, corresponding to the intermediate tertile. (3) An ‘advanced’ group, made up of participants with brain-PAD values in the highest tertile (Chakrabarty et al., 2023).

## 3 Results

### 3.1 Demographic characteristics

Fifty-seven patients with chronic left hemisphere PSA (38 male, 19 female) were included. The mean disease duration was 19.16 ± 18.54 months. The mean WAB-AQ was 62.13 ± 17.30. Detailed demographic and clinical characteristics are presented in Table 1.

**Table 1:** Demographic and clinical characteristics of patients with PSA.

Variable	Patients with PSA (*n* = 57)
Demographic characteristics	
Age (years), mean (SD)	55.04 (9.01)
Education (years), mean (SD)	9.63 (2.75)
Gender (male), *n* (%)	38 (66.67%)
Handness (right), *n* (%)	57 (100.00%)
Hpertension, *n* (%)	41 (71.93%)
Diabetes mellitus, *n* (%)	20 (35.09%)
Clinical information	
Time since stroke (months), mean (SD)	19.16 (18.54)
Lesion volume (mL), mean (SD)	69.18 (105.54)
WMH severity, mean (SD)	
PWMH	1.46 (0.68)
DWMH	1.51 (0.73)
WAB, mean (SD)	
AQ	62.13 (17.30)
Spontaneous speech	24.18 (9.90)
Auditory comprehension	12.92 (4.93)
Repetition	12.09 (4.94)
Naming	12.94 (5.65)
Brain age (years), mean (SD)	
Brain age	57.23 (10.73)
Brain-PAD	2.20 (6.45)

Data are presented as mean ± standard deviation or number (percentage). AQ, Aphasia Quotient; β, standardized coefficients; brain-PAD, Brain-Predicted Age Difference; DWMH, Deep White Matter Hyperintensities; PSA, Post-Stroke Aphasia; PWMH, Periventricular White Matter Hyperintensities; *q*, FDR-adjusted p value; WAB, Western Aphasia Battery.

### 3.2 Brain-PAD

**Figure 2:**
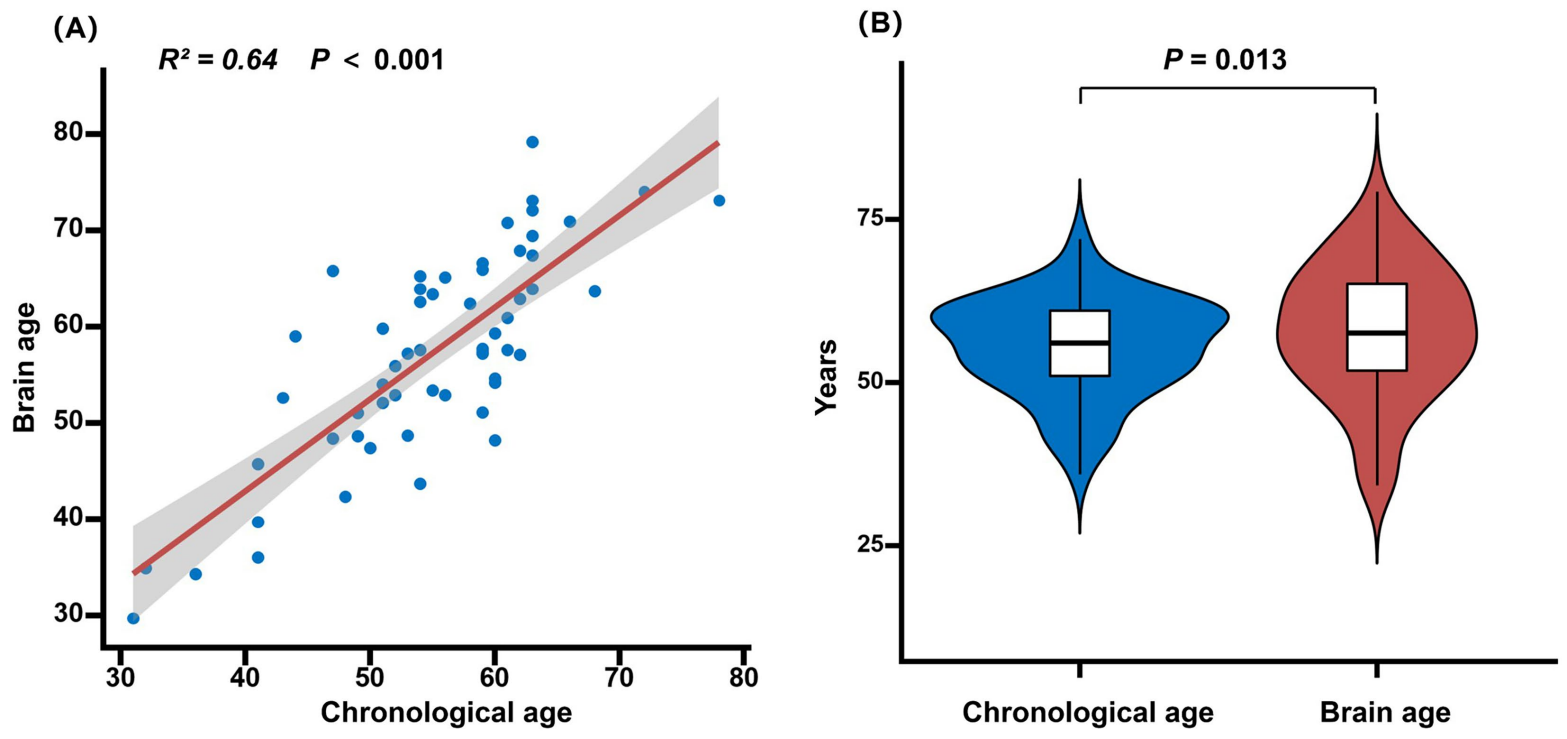
Correlation between brain age and chronological age in PSA. **(A)** Positive correlation between brain age and chronological age (shaded bands represent 95% confidence intervals). **(B)** Brain age is significantly older than chronological age in PSA patients. PSA, Post-Stroke Aphasia.

Patients’ predicted brain age ranged from 11.8 years younger to 18.8 years older than their chronological age. The mean brain age in patients with PSA was 57.23 ± 10.73 years, and the mean brain-PAD was 2.20 ± 6.45 years. A significant positive correlation was observed between chronological age and estimated brain age (*R*^2^ = 0.64, *p* < 0.001). Mean estimated brain age was significantly older than chronological age (*p* = 0.013) (Figure 2).

### 3.3 Relationship between brain-PAD and WMH

The effects of PWMH and DWMH on brain-PAD were examined via linear regression analyses (Supplementary Figure 1). In a model with PWMH scores as the independent variable, PWMH was significantly associated with increased brain-PAD (*β* = 3.217, *p* = 0.024). DWMH was significantly positively associated with brain-PAD (*β* = 5.872, *p* < 0.001).

### 3.4 Mediation analysis of WMH, brain-PAD, and aphasia severity

Prior to mediation analysis, the associations between WMH burden and aphasia severity were assessed using multivariate linear regression. PWMH or DWMH burden was treated as the independent variable, and the WAB subscores were used as dependent variables. The models adjusted for potential confounders, including chronological age, sex, education level, hypertension, diabetes, lesion volume, and time since stroke. Preliminary analyses indicated that PWMH significantly affected the auditory comprehension subscore (*β* = −36.507, *p* = 0.001, *q* = 0.004), and naming subscore (*β* = −17.429, *p* = 0.008, q = 0.017). Similarly, DWMH significantly affected the auditory comprehension subscore (*β* = −49.703, *p* < 0.001, *q* = 0.001), and naming subscore (*β* = −20.545, *p* = 0.009, *q* = 0.017). No significant associations were observed with the spontaneous speech or repetition subscores. Therefore, these were excluded from subsequent mediation analysis (Supplementary Table 2).

**Figure 3:**
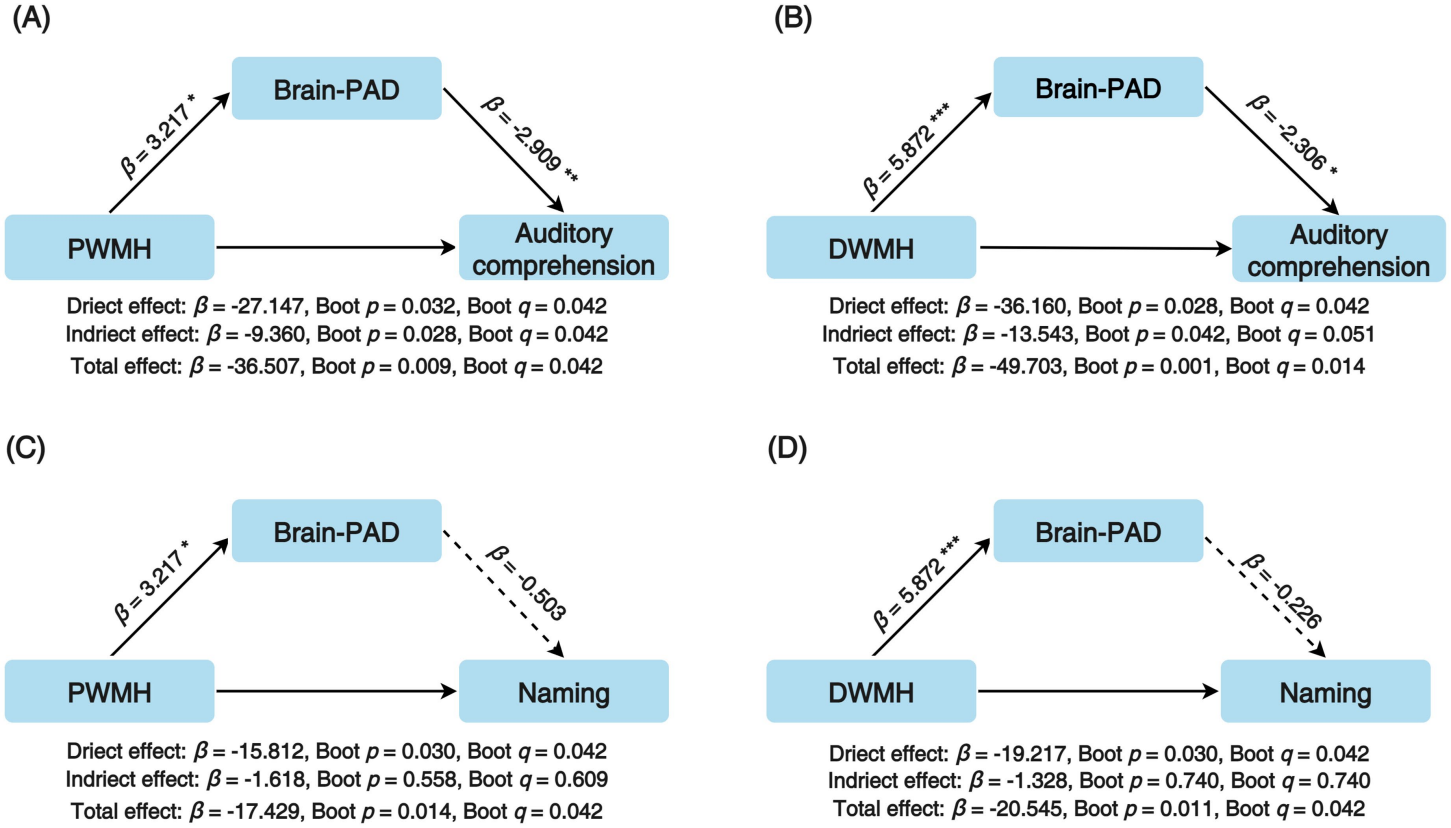
Mediation analysis for WAB subscores in PSA. **(A)** PWMH burden as the independent variable and auditory comprehension score as the dependent variable. **(B)** DWMH burden as the independent variable and auditory comprehension score as the dependent variable. **(C)** PWMH burden as the independent variable and naming score as the dependent variable; **(D)** DWMH burden as the independent variable and naming score as the dependent variable. *β*, standardized coefficients; brain-PAD, Brain-Predicted Age Difference; DWMH, Deep White Matter Hyperintensities; PWMH, Periventricular White Matter Hyperintensities; *q*, FDR-adjusted *p* value; WAB, Western Aphasia Battery.

Brain-PAD was included as an additional covariate to examine its mediating role in the relationship between WMH burden and aphasia severity. The indirect effect of PWMH on the auditory comprehension subscores, mediated by brain-PAD, was statistically significant (*β* = −9.360, *p* = 0.028, *q* = 0.042). The proportion of mediated effect accounted for 25.64% of the total effect (*p* = 0.034). Notably, the direct effect of PWMH on the auditory comprehension subscores remained statistically significant (*β* = −27.147, *p* = 0.032, *q* = 0.042). Naming subscore was directly affected by both PWMH (*β* = −15.812, *p* = 0.030, *q* = 0.042) and DWMH (*β* = −19.217, *p* = 0.030, *q* = 0.042), no significant indirect effects were observed (Figure 3).

### 3.5 Interaction analysis between WMH and brain-PAD

We revealed a significant interaction between PWMH burden and brain-PAD on the auditory comprehension subscore (*β* = −4.040, *p* = 0.004, *q* = 0.033, Cohen *f ^2^* = 0.20), whereas no significant interactive effect between DWMH and brain-PAD was observed for any subscore of theWAB (all *q* > 0.05; Table 2).

**Table 2:** Interaction effects between PWMH and brain-PAD on auditory comprehension.

WAB	Interaction analysis between brain-PAD and WMH					
	PWMH			DWMH		
	*β*	*p*	*q*	*β*	*p*	*q*
Spontaneous speech	0.048	0.771	0.771	0.053	0.726	0.771
Auditory comprehension	−4.040	0.004	**0.033**	0.868	0.523	0.771
Repetition	0.236	0.771	0.771	0.318	0.673	0.771
Naming	−0.352	0.715	0.771	−1.506	0.091	0.363

Bold values indicate significant FDR-adjusted *p* value. *β*, standardized coefficients; brain-PAD, Brain-Predicted Age Difference; DWMH, Deep White Matter Hyperintensities; PWMH, Periventricular White Matter Hyperintensities; *q*, FDR-adjusted *p* value; WAB, Western Aphasia Battery.

**Figure 4:**
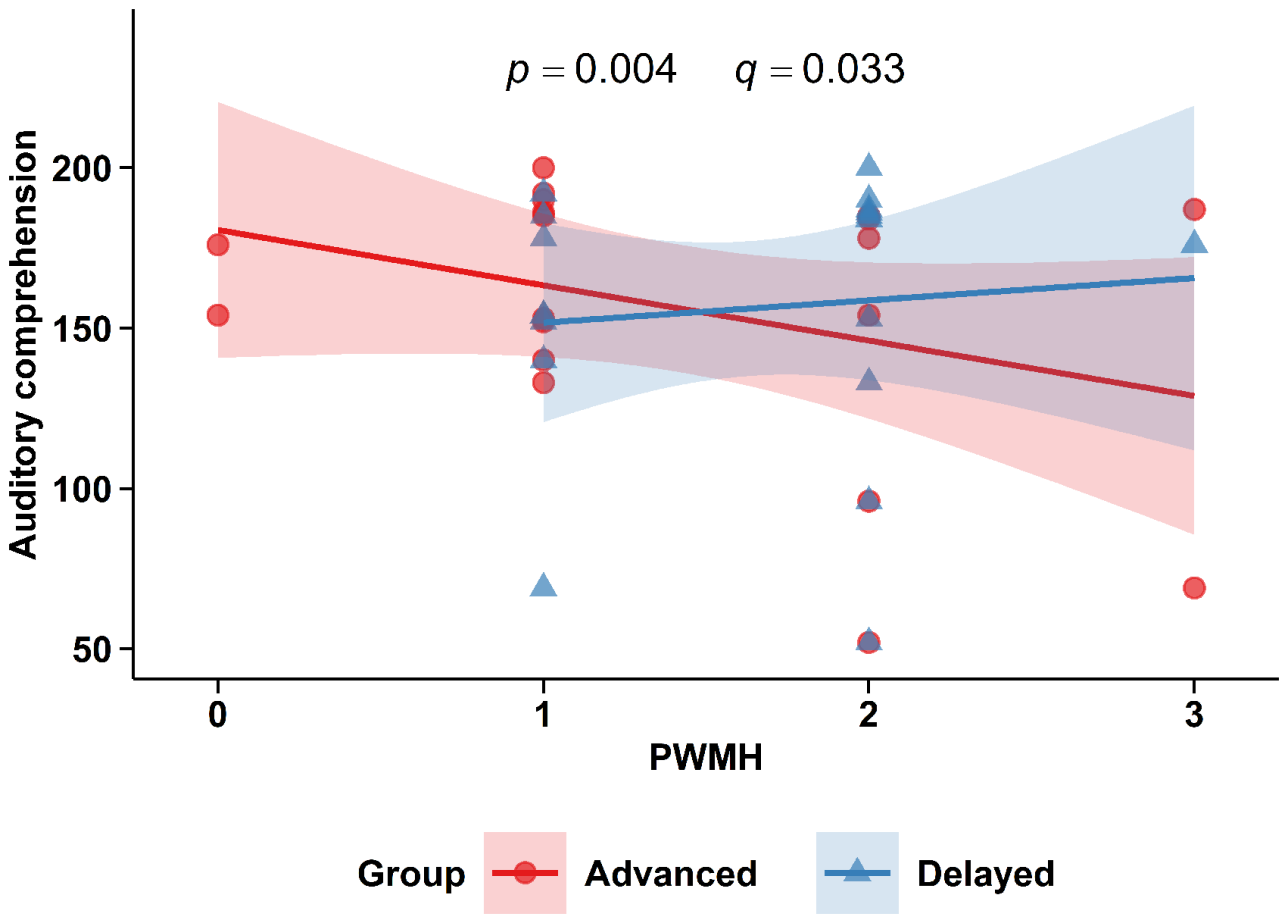
Interaction analysis between PWMH burden and brain-PAD on auditory comprehension. Brain-PAD, Brain-Predicted Age Difference; PWMH, Periventricular WMH.

In the group with brain-PAD in the highest tertile (range: 4.7–18.8 years), which corresponds to an “advanced” brain aging phenotype, auditory comprehension demonstrated a decreasing trend as the PWMH increased. In contrast, in the group with brain-PAD in the lowest tertile (range: −11.8 to −1.3 years), which corresponds to a “delayed” brain aging phenotype, auditory comprehension exhibited a slight increasing trend with escalating PWMH (Figure 4).

## 4 Discussion

This study investigated the relationship between WMH burden and brain-PAD, as well as their combined effects on language deficits in patients with PSA. The results demonstrated that brain-PAD was substantially influenced by both PWMH and DWMH burden. The effects of PWMH on the auditory comprehension subscore were indirectly mediated by brain-PAD, whereas the naming subscore was directly affected by WMH. Additionally, an interaction between brain-PAD and PWMH was observed in auditory comprehension.

Multivariate regression analysis showed that both PWMH and DWMH were associated with increased brain-PAD. This association may result from WMH - related hemodynamic abnormalities. These abnormalities impair cerebral perfusion, induce chronic hypoxia, and reduce nutrient delivery, and these factors may accelerate brain aging (Lambert et al., 2016). WMH-induced neuroinflammation and oxidative stress can also damage neuronal integrity and further exacerbate brain aging (Fernando et al., 2006). Disruption of the blood–brain barrier may allow harmful substances to infiltrate brain tissue, whereas impairments in axonal transport can reduce neurotrophic factor delivery, contributing to progressive neuronal dysfunction (van Gijn, 1998; Wardlaw et al., 2003, 2013; Wang et al., 2023). As PWMH and DWMH accumulate, they may exert widespread effects across brain regions, thereby increasing brain age and accelerating.

Mediation analysis further clarified how WMH contributes to post-stroke language impairment, revealing symptom specific effects. Although prior studies have linked WMH severity with naming difficulties (Wang et al., 2013; Wright et al., 2018), the association with auditory comprehension has remained less defined. The present findings suggest that the effect of PWMH on auditory comprehension is partially mediated by brain-PAD. Auditory comprehension depends on the coordinated activity of multiple brain regions. PWMH may disrupt this network by accelerating brain tissue aging, reducing the efficiency of interregional information transfer, and weakening functional connectivity (Langen et al., 2018). Our study also showed that WMH has a direct effect on naming performance, consistent with previous findings (Wright et al., 2018). Collectively, these results underscore the differential impact of WMH on auditory comprehension and naming in PSA and highlight brain-PAD as a critical factor for evaluating and managing post-stroke language deficits, particularly those related to auditory comprehension.

This study also identified an interaction between PWMH burden and brain-PAD in auditory comprehension subscores. Specifically, the impact of PWMH on auditory comprehension was more pronounced in the advanced brain aging patients, suggesting that advanced brain aging amplifies these detrimental effects. Increased vulnerability of the aging brain to neurological disorders likely reduces the efficiency of cellular maintenance and repair processes. Furthermore, diminished repair capacity may render the brain more susceptible to pathological changes such as WMH, thereby contributing to more pronounced deficits in cognitive functions, including auditory comprehension (Cole et al., 2019; Wrigglesworth et al., 2021). Consequently, the interaction between PWMH and brain-PAD may further impede language recovery in PSA by exacerbating dysfunction in already compromised neural networks.

This study had some limitations. First, the use of a single-center design and a relatively small sample size. Future studies should involve larger cohorts to validate the stability and generalizability of the findings. Second, although cross-sectional studies provide valuable insights, longitudinal studies are better suited to characterize the progression of PSA from the acute to the chronic stage. Third, the study relied solely on structural neuroimaging data, and additional neuroimaging modalities such as diffusion tensor imaging (DTI) were not incorporated. Third, we did not collect stroke etiology and acute treatment strategies. Though hypertension and diabetes were controlled as covariates, the absence of detailed data is a potential residual confounder. Future studies should include these to reduce confounding and improve result specificity. Finally, although the Fazekas scale is widely used due to its high inter-rater reliability and strong correlation with WMH volume, it does not provide precise quantitative data. Future investigations may benefit from WMH quantification using techniques such as deep learning-based U-Net image segmentation (Fei et al., 2024).

## 5 Conclusion

Our study demonstrates that brain-PAD serves as a significant mediator in the relationship between PWMH and auditory comprehension. Additionally, the naming subscore is directly influenced by both PWMH and DWMH. Notably, the impact of PWMH on auditory comprehension is more pronounced in patients with advanced brain aging. Collectively, these findings highlight a close association between WMHs, brain-PAD, and the severity of aphasia in patients with PSA.

## Data Availability

The raw data supporting the conclusions of this article will be made available by the authors, without undue reservation.

## Footnotes

^1^ https://www.slicer.org

## References

[ref1] Abram S. V. Roach B. J. Hua J. P. Y. Han L. K. M. Mathalon D. H. Ford J. M.. (2023). Advanced brain age correlates with greater rumination and less mindfulness in schizophrenia. Neuroimage Clin. 37:103301. doi: 10.1016/j.nicl.2022.103301 36586360 PMC9830317

[ref2] Baecker L. Garcia-Dias R. Vieira S. Scarpazza C. Mechelli A. (2021). Machine learning for brain age prediction: introduction to methods and clinical applications. EBioMedicine 72:103600. doi: 10.1016/j.ebiom.2021.103600 34614461 PMC8498228

[ref3] Baron R. M. Kenny D. A. (1986). The moderator–mediator variable distinction in social psychological research: conceptual, strategic, and statistical considerations. J. Pers. Soc. Psychol. 51, 1173–1182. doi: 10.1037/0022-3514.51.6.1173 3806354

[ref4] Basilakos A. Stark B. C. Johnson L. Rorden C. Yourganov G. Bonilha L.. (2019). Leukoaraiosis is associated with a decline in language abilities in chronic aphasia. Neurorehabil. Neural Repair 33, 718–729. doi: 10.1177/1545968319862561 31315507 PMC6693961

[ref5] Brickman A. M. Siedlecki K. L. Muraskin J. Manly J. J. Luchsinger J. A. Yeung L.-K.. (2011). White matter hyperintensities and cognition: testing the reserve hypothesis. Neurobiol. Aging 32, 1588–1598. doi: 10.1016/j.neurobiolaging.2009.10.013 19926168 PMC2891625

[ref6] Busby N. Newman-Norlund S. Sayers S. Newman-Norlund R. Wilson S. Nemati S.. (2022). White matter hyperintensity load is associated with premature brain aging. Aging (Albany NY) 14, 9458–9465. doi: 10.18632/aging.204397 36455869 PMC9792198

[ref7] Chakrabarty T. Frangou S. Torres I. J. Ge R. Yatham L. N. (2023). Brain age and cognitive functioning in first-episode bipolar disorder. Psychol. Med. 53, 5127–5135. doi: 10.1017/S0033291722002136 35875930 PMC10476063

[ref8] Chen Y.-S. Kuo C.-Y. Lu C.-H. Wang Y.-W. Chou K.-H. Lin W.-C. (2024). Multiscale brain age prediction reveals region-specific accelerated brain aging in Parkinson’s disease. Neurobiol. Aging 140, 122–129. doi: 10.1016/j.neurobiolaging.2024.05.003 38776615

[ref9] Cohen J. (2009). Statistical power analysis for the behavioral sciences. 2nd Edn. New York, NY: Psychology Press.

[ref10] Cole J. H. Franke K. (2017). Predicting age using neuroimaging: innovative brain ageing biomarkers. Trends Neurosci. 40, 681–690. doi: 10.1016/j.tins.2017.10.001 29074032

[ref11] Cole J. H. Marioni R. E. Harris S. E. Deary I. J. (2019). Brain age and other bodily “ages”: implications for neuropsychiatry. Mol. Psychiatry 24, 266–281. doi: 10.1038/s41380-018-0098-1 29892055 PMC6344374

[ref12] Cole J. H. Poudel R. P. K. Tsagkrasoulis D. Caan M. W. A. Steves C. Spector T. D.. (2017). Predicting brain age with deep learning from raw imaging data results in a reliable and heritable biomarker. NeuroImage 163, 115–124. doi: 10.1016/j.neuroimage.2017.07.059 28765056

[ref13] Cole J. H. Ritchie S. J. Bastin M. E. Valdés Hernández M. C. Muñoz Maniega S. Royle N.. (2018). Brain age predicts mortality. Mol. Psychiatry 23, 1385–1392. doi: 10.1038/mp.2017.62 28439103 PMC5984097

[ref14] Dalby R. B. Chakravarty M. M. Ahdidan J. Sørensen L. Frandsen J. Jonsdottir K. Y.. (2010). Localization of white-matter lesions and effect of vascular risk factors in late-onset major depression. Psychol. Med. 40, 1389–1399. doi: 10.1017/S0033291709991656 19895719

[ref15] de Leeuw F. E. de Groot J. C. Achten E. Oudkerk M. Ramos L. M. Heijboer R.. (2001). Prevalence of cerebral white matter lesions in elderly people: a population based magnetic resonance imaging study. The Rotterdam scan study. J. Neurol. Neurosurg. Psychiatry 70, 9–14. doi: 10.1136/jnnp.70.1.9 11118240 PMC1763449

[ref16] Fazekas F. Chawluk J. B. Alavi A. Hurtig H. I. Zimmerman R. A. (1987). MR signal abnormalities at 1.5 T in Alzheimer’s dementia and normal aging. AJR Am. J. Roentgenol. 149, 351–356. doi: 10.2214/ajr.149.2.351 3496763

[ref17] Fei B. Cheng Y. Liu Y. Zhang G. Ge A. Luo J.. (2024). Intelligent cholinergic white matter pathways algorithm based on U-net reflects cognitive impairment in patients with silent cerebrovascular disease. Stroke Vasc. Neurol. 9, 699–707. doi: 10.1136/svn-2023-002976 38569895 PMC11791635

[ref18] Fernando M. S. Simpson J. E. Matthews F. Brayne C. Lewis C. E. Barber R.. (2006). White matter lesions in an unselected cohort of the elderly: molecular pathology suggests origin from chronic hypoperfusion injury. Stroke 37, 1391–1398. doi: 10.1161/01.STR.0000221308.94473.14 16627790

[ref19] Garde E. Mortensen E. L. Krabbe K. Rostrup E. Larsson H. B. (2000). Relation between age-related decline in intelligence and cerebral white-matter hyperintensities in healthy octogenarians: a longitudinal study. Lancet 356, 628–634. doi: 10.1016/S0140-6736(00)02604-0 10968435

[ref20] Griswold M. A. Jakob P. M. Heidemann R. M. Nittka M. Jellus V. Wang J.. (2002). Generalized autocalibrating partially parallel acquisitions (GRAPPA). Magn. Reson. Med. 47, 1202–1210. doi: 10.1002/mrm.10171 12111967

[ref21] Kim K. W. MacFall J. R. Payne M. E. (2008). Classification of white matter lesions on magnetic resonance imaging in elderly persons. Biol. Psychiatry 64, 273–280. doi: 10.1016/j.biopsych.2008.03.024 18471801 PMC2593803

[ref22] Koo T. K. Li M. Y. (2016). A guideline of selecting and reporting Intraclass correlation coefficients for reliability research. J. Chiropr. Med. 15, 155–163. doi: 10.1016/j.jcm.2016.02.012 27330520 PMC4913118

[ref23] Kristinsson S. Zhang W. Rorden C. Newman-Norlund R. Basilakos A. Bonilha L.. (2021). Machine learning-based multimodal prediction of language outcomes in chronic aphasia. Hum. Brain Mapp. 42, 1682–1698. doi: 10.1002/hbm.25321 33377592 PMC7978124

[ref24] Lambert C. Benjamin P. Zeestraten E. Lawrence A. J. Barrick T. R. Markus H. S. (2016). Longitudinal patterns of leukoaraiosis and brain atrophy in symptomatic small vessel disease. Brain 139, 1136–1151. doi: 10.1093/brain/aww009 26936939 PMC4806220

[ref25] Langen C. D. Cremers L. G. de Groot M. White T. Ikram M. A. Niessen W. J.. (2018). Disconnection due to white matter hyperintensities is associated with lower cognitive scores. NeuroImage 183, 745–756. doi: 10.1016/j.neuroimage.2018.08.037 30144572

[ref26] Lau K. K. Li L. Schulz U. Simoni M. Chan K. H. Ho S. L.. (2017). Total small vessel disease score and risk of recurrent stroke: validation in 2 large cohorts. Neurology 88, 2260–2267. doi: 10.1212/WNL.0000000000004042 28515266 PMC5567324

[ref27] Lee J. Burkett B. J. Min H.-K. Senjem M. L. Lundt E. S. Botha H.. (2022). Deep learning-based brain age prediction in normal aging and dementia. Nat. Aging 2, 412–424. doi: 10.1038/s43587-022-00219-7 37118071 PMC10154042

[ref28] Li H. Zhang H. Xu S. Wang M. Zhang J. Liu J.. (2023). Altered spontaneous brain activity in poststroke aphasia: a resting-state fMRI study. Brain Sci. 13:300. doi: 10.3390/brainsci13020300 36831843 PMC9954170

[ref29] Lin Q. Huang W.-Q. Ma Q.-L. Lu C.-X. Tong S.-J. Ye J.-H.. (2017). Incidence and risk factors of leukoaraiosis from 4683 hospitalized patients: a cross-sectional study. Medicine (Baltimore) 96:e7682. doi: 10.1097/MD.0000000000007682 28953609 PMC5626252

[ref30] Longstreth W. T. Manolio T. A. Arnold A. Burke G. L. Bryan N. Jungreis C. A.. (1996). Clinical correlates of white matter findings on cranial magnetic resonance imaging of 3301 elderly people: the cardiovascular health study. Stroke 27, 1274–1282. doi: 10.1161/01.str.27.8.1274 8711786

[ref31] Radwan A. M. Emsell L. Blommaert J. Zhylka A. Kovacs S. Theys T.. (2021). Virtual brain grafting: enabling whole brain parcellation in the presence of large lesions. NeuroImage 229:117731. doi: 10.1016/j.neuroimage.2021.117731 33454411

[ref32] Salvalaggio A. De Filippo De Grazia M. Zorzi M. Thiebaut de Schotten M. Corbetta M. (2020). Post-stroke deficit prediction from lesion and indirect structural and functional disconnection. Brain 143, 2173–2188. doi: 10.1093/brain/awaa156 32572442 PMC7363494

[ref33] Shewan C. M. Kertesz A. (1980). Reliability and validity characteristics of the Western aphasia battery (WAB). J. Speech Hear. Disord. 45, 308–324. doi: 10.1044/jshd.4503.308 7412225

[ref34] Vadinova V. Sihvonen A. J. Garden K. L. Ziraldo L. Roxbury T. O’Brien K.. (2023). Hyperintensities and recovery of language after stroke. Neurorehabil. Neural Repair 37, 218–227. doi: 10.1177/15459683231168384 37083133 PMC10152219

[ref35] van Gijn J. (1998). Leukoaraiosis and vascular dementia. Neurology 51, S3–S8. doi: 10.1212/wnl.51.3_suppl_3.s3 9744823

[ref36] Van Leijsen E. M. C. Van Uden I. W. M. Ghafoorian M. Bergkamp M. I. Lohner V. Kooijmans E. C. M.. (2017). Nonlinear temporal dynamics of cerebral small vessel disease: the RUN DMC study. Neurology 89, 1569–1577. doi: 10.1212/WNL.0000000000004490 28878046 PMC5634663

[ref37] Wang J. Marchina S. Norton A. C. Wan C. Y. Schlaug G. (2013). Predicting speech fluency and naming abilities in aphasic patients. Front. Hum. Neurosci. 7:831. doi: 10.3389/fnhum.2013.00831 24339811 PMC3857577

[ref38] Wang X. Shi Y. Chen Y. Gao Y. Wang T. Li Z.. (2023). Blood–brain barrier breakdown is a sensitive biomarker of cognitive and language impairment in patients with White matter Hyperintensities. Neurol. Ther. 12, 1745–1758. doi: 10.1007/s40120-023-00527-z 37490234 PMC10444912

[ref39] Wardlaw J. M. Doubal F. N. Valdes-Hernandez M. Wang X. Chappell F. M. Shuler K.. (2013). Blood–brain barrier permeability and long-term clinical and imaging outcomes in cerebral small vessel disease. Stroke 44, 525–527. doi: 10.1161/STROKEAHA.112.669994 23233386 PMC3843346

[ref40] Wardlaw J. M. Sandercock P. A. G. Dennis M. S. Starr J. (2003). Is breakdown of the blood-brain barrier responsible for lacunar stroke, Leukoaraiosis, and dementia? Stroke 34, 806–812. doi: 10.1161/01.STR.0000058480.77236.B3 12624314

[ref41] Wrigglesworth J. Ward P. Harding I. H. Nilaweera D. Wu Z. Woods R. L.. (2021). Factors associated with brain ageing - a systematic review. BMC Neurol. 21:312. doi: 10.1186/s12883-021-02331-4 34384369 PMC8359541

[ref42] Wrigglesworth J. Yaacob N. Ward P. Woods R. L. McNeil J. Storey E.. (2022). Brain-predicted age difference is associated with cognitive processing in later-life. Neurobiol. Aging 109, 195–203. doi: 10.1016/j.neurobiolaging.2021.10.007 34775210 PMC8832483

[ref43] Wright A. Tippett D. Saxena S. Sebastian R. Breining B. Faria A.. (2018). Leukoaraiosis is independently associated with naming outcome in poststroke aphasia. Neurology 91, e526–e532. doi: 10.1212/WNL.0000000000005945 29980639 PMC6105047

[ref44] Yourganov G. Fridriksson J. Rorden C. Gleichgerrcht E. Bonilha L. (2016). Multivariate connectome-based symptom mapping in post-stroke patients: networks supporting language and speech. J. Neurosci. 36, 6668–6679. doi: 10.1523/JNEUROSCI.4396-15.2016 27335399 PMC4916245

[ref45] Zhu R. Qu J. Xu G. Wu Y. Xin J. Wang D. (2024). Clinical and multimodal imaging features of adult-onset neuronal intranuclear inclusion disease. Neurol. Sci. 45, 5795–5805. doi: 10.1007/s10072-024-07699-y 39023713 PMC11554744

