# Supplementary material for: Mediating role of brain aging in the effect of white matter hyperintensities on post-stroke aphasia severity
Source: Front Aging Neurosci. 2025 Oct 16;17:1629870. doi: 10.3389/fnagi.2025.1629870 (PMC12571856; doi:10.3389/fnagi.2025.1629870)
Supplement: Supplementary file 4 [file Table_4.docx]

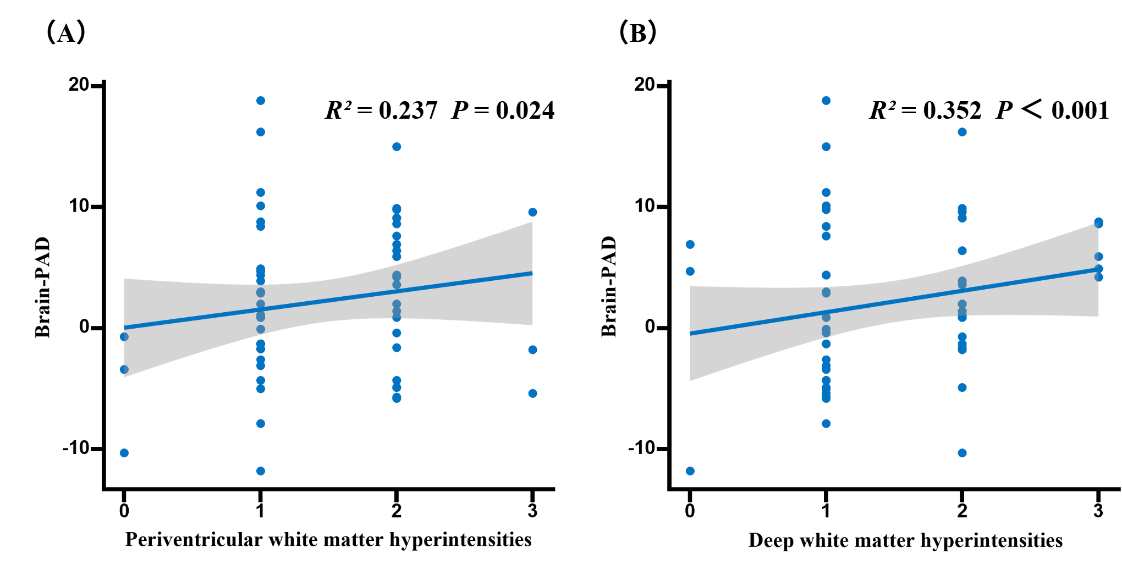


**Supplementary Figure 1.** Association between PWMH/DWMH burden and brain-PAD. Shaded bands represent 95% confidence intervals. brain-PAD, brain-predicted age difference; DWMH, deep white matter hyperintensity; PWMH, periventricular white matter hyperintensity.

**Supplementary Table 1**. Inter-rater reliability of Fazekas scores for PWMH and DWMH.

| **WMH Type** | **ICC** | **95% CI** | ***p*** |
| --- | --- | --- | --- |
| PWMH | 0.943 | 0.906, 0.967 | <0.001 |
| DWMH | 0.928 | 0.882, 0.958 | <0.001 |

ICC: The inter-rater reliability was evaluated using the two-way random effects ICC for absolute agreement. ICC values were interpreted as follows: >0.75: excellent agreement; 0.60–0.75: good agreement; 0.40–0.60: moderate agreement; <0.40: poor agreement. Abbreviations: DWMH, deep white matter hyperintensity; ICC, Intraclass Correlation Coefficient;PWMH, periventricular white matter hyperintensity; WMH, white matter hyperintensity.

**Supplementary Table 2.** Multivariate linear regression analyses for WAB subscores

|  |  | Before adjust brain-PAD | | | After adjust brain-PAD | | |
| --- | --- | --- | --- | --- | --- | --- | --- |
|  |  | *β* | *p* | *q* | *β* | *p* | *q* |
| Spontaneous speech | PWMH | 0.017 | 0.987 | 0.987 | 0.380 | 0.737 | 0.976 |
|  | DWMH | -0.625 | 0.625 | 0.899 | -0.044 | 0.976 | 0.976 |
| Auditory comprehension | PWMH | -36.507 | 0.001 | **0.004** | -27.147 | 0.010 | **0.040** |
|  | DWMH | -49.703 | <0.001 | **0.001** | -36.160 | 0.008 | **0.040** |
| Repetition | PWMH | -0.190 | 0.972 | 0.987 | 1.635 | 0.771 | 0.976 |
|  | DWMH | -2.666 | 0.674 | 0.899 | 0.473 | 0.948 | 0.976 |
| Naming | PWMH | -17.429 | 0.008 | **0.017** | -15.812 | 0.021 | 0.057 |
|  | DWMH | -20.545 | 0.009 | **0.017** | -19.217 | 0.032 | 0.063 |

Multivariate linear regression models were used to investigate the association between WAB subscores and WMHs, while adjusting for age, sex, education, hypertension, diabetes, lesion volume, and time post-stroke and/or brain-PAD as covariates. Abbreviations: *β*, standardized beta coefficient; brain-PAD, brain-predicted age difference; DWMH, deep white matter hyperintensity; PWMH, periventricular white matter hyperintensity; *q*, false discovery rate-adjusted P-value; WAB, Western Aphasia Battery; WMH, white matter hyperintensity.
